# *Aronia melanocarpa* Ameliorates Adrenal Cytoarchitecture Against the Hexavalent Chromium-Induced Injury

**DOI:** 10.1007/s12011-020-02401-7

**Published:** 2020-10-01

**Authors:** Jelena Savici, Romeo Teodor Cristina, Diana Brezovan, Isidora Radulov, Cornel Balta, Oana Maria Boldura, Florin Muselin

**Affiliations:** 1 Faculty of Veterinary Medicine, Department of Histology and Cell biology, Banat University of Agriculture and Veterinary Medicine “King Michael I of Romania” from Timisoara (BUAVM), Calea Aradului 119, 300645 Timișoara, Romania; 2 Faculty of Veterinary Medicine, Department of Pharmacoogy and Pharmacy, Banat University of Agriculture and Veterinary Medicine “King Michael I of Romania” from Timisoara (BUAVM), Calea Aradului 119, 300645 Timișoara, Romania; 3 Faculty of Veterinary Medicine, Department of Chemistry, Banat University of Agriculture and Veterinary Medicine “King Michael I of Romania” from Timisoara (BUAVM), Calea Aradului 119, 300645 Timișoara, Romania; 4 “Vasile Goldis” Western University Arad, Revolutiei Blvd. No, 94 Arad, Romania; 5 Faculty of Veterinary Medicine, Department of Biochemistry, Banat University of Agriculture and Veterinary Medicine “King Michael I of Romania” from Timisoara (BUAVM), Calea Aradului 119, 300645 Timișoara, Romania; 6 Faculty of Veterinary Medicine, Department of Toxicology, Banat University of Agriculture and Veterinary Medicine “King Michael I of Romania” from Timisoara (BUAVM), Calea Aradului 119, 300645 Timișoara, Romania

**Keywords:** Adrenal gland, Hexavalent chromium, *Aronia melanocarpa*, *Bax/Bcl2* genes, Cytoarchitecture

## Abstract

Hexavalent chromium is a toxin that penetrates the cell, triggering reactive oxygen species (ROS) production. *Aronia melanocarpa*, due to its proanthocyanidins, anthocyanins, and phenolic acid contents, is a valuable antioxidant. The aim was to observe the influence of hexavalent chromium Cr(VI) on the adrenal gland, and if this impact can be recovered by the administration of *A. melanocarpa.* Accordingly, 36 rats were divided into six groups: *control; Aronia*; *Cr* receiving Cr(VI) in distilled water for 3 months; *CrA* receiving a mix of Cr(VI) and *A. melanocarpa* at 2.5% aqueous extract for 3 months; *Cr2* receiving, for 3 months, Cr(VI) in distilled water, and next, for 1 month, only distilled water; and respectively, *CrA2* receiving, for 3 months, Cr(VI) in distilled water, followed by 1 month of *Aronia* at 2.5% extract administration. The adrenal gland samples were examined toward histological and molecular assessment, and results were statistically analyzed (ANOVA). Hexavalent chromium induced changes in the adrenal cortex expressed by focal or diffuse hypertrophies, cytoplasmic vacuolization (due to lipidic accumulation), and cells’ shape and size alteration, including necrosis. These structural alterations were carried by *Bax* and *Bcl2* gene expression: the *Bax* gene expression levels, increased significantly (*p* < 0.001) in all experimental groups, except the *Aronia* group, compared with control. In the *Cr2*, *CrA*, and *CrA2* groups, notable reduction of *Bax* gene expression (*p* < 0.001) was reported compared with the *Cr* group. Regarding the *Bcl2* gene expression (*p* < 0.001), a significant increase was observed in the experimental groups, compared with the control. The *Bcl2* expression level had a similar pattern to *Bax* gene, consequently trying to compensate its overexpression. *Aronia* administered concomitantly, or after Cr(VI), diminished structural changes and expression of the studied genes, thus reducing the *Bax/Bcl2* ratio and suggesting that the active ingredients from *Aronia* are capable of blocking apoptotic cascade induced by the pathway of *Bax* and *Bcl2* proteins.

## Introduction

Due to its anthropogenic factor, chromium is considered an environmental pollutant [1]. Hexavalent chromium is reasonably related to the structure of sulfate ions, which makes it possible for chromium to enter into the cell, within the sulfate channels of the cell membrane. Once inside the cell, oxidant properties of chromium are displayed, since the reduction of hexavalent to trivalent form is a chain reaction with the production of reactive oxygen species (ROS) and intermediates; Cr(V) and Cr(IV) [2].

Consequently, oxidative stress occurs by ROS formation during Cr(VI) reduction or, due to oxidizing properties, of Cr(V) intermediate [3]. The ROS generated are superoxide, hydroxyl, and hydrogen peroxide, all sources for hydroxyl radicals [4].

Due to natural existence and human activity, hexavalent chromium is ubiquitous in air, soil, and water. Hence, people and animals can be exposed by breathing, swallowing, drinking, and by skin contact with chromium compounds. Studies have shown that hexavalent chromium is responsible for inducing negative respiratory, gastrointestinal, renal, cardiovascular, hepatic, carcinogenic and mutagenic, hematological, reproductive, and neurological effects. In this reason, the world is more oriented toward new phytotherapeutic means to attenuate the deleterious activity of Cr(VI) [5].

*Aronia melanocarpa* (*Rosaceae) L*. is abundant in proanthocyanidins, anthocyanins, and phenolic acid, possessing the highest antioxidant activity among the plants [6]. This is expressed by radical scavenging, nitrogen inhibition, oxygen species formation, restoration of antioxidant enzymes’ level, and prooxidant enzymes’ suppression [7].

Meng et al. presented that *A. melanocarpa* anthocyanins have the ability to inhibit mitochondrial dysfunction by increasing the expression of *Bcl2* protein and decreasing the expression of *Bax* protein, and thus attenuating apoptosis in SH-SY5Y cell line [8].

Proteins forming *Bcl2* family are responsible for the regulation of apoptosis by mitochondrial pathways [9]. *Bax* protein, a proapoptotic member of the *Bcl2* family, is accountable for the appearance of pores in the mitochondrial membrane and the release of cytochrome *c*, followed by the activation of caspase cascade promoting apoptosis [10]. *Bcl2* protein is an antiapoptotic factor that can interact with *Bax* proteins, overturning their reaction, and inhibiting the changes in the mitochondrial membrane [9].

The *Bax/Bcl2* ratio regulates the execution phase of apoptosis, functionally controlling the mitochondria. Excess of *Bcl2* protein (antiapoptotic) ensures cell survival, either by directly blocking the action of *Bax* protein or by preventing the activation of caspases—the main enzymes involved in apoptosis—influencing mitochondrial membrane permeability [2]. Excess of *Bax* protein (proapoptotic) leads to the inactivation of *Bcl2* and changes in mitochondrial transmembrane potential through the formation of transmembrane complexes. As a result, exchanges between the mitochondrial compartment and the cytoplasm will intensify, leading to the release of cytochrome *c* proteins, second mitochondria-derived activator of caspases/Diablo homolog (SMAC/DIABLO), which will generate caspase activation and trigger the apoptotic cascade [11].

Banu et al. showed that chromium treatment in vitro of granulosa cells, harvested from female rats, induced increase in *Bax* gene expression and decrease of *Bcl2* gene expression and hence apoptotic cell death [12]. Similar results of gene expression leading to apoptosis were obtained on the skin fibroblasts of an Indo-Pacific humpback dolphin exposed to hexavalent chromium [13].

However, Marouani et al. conducted an in vivo study on rats exposed intra-peritoneally to potassium dichromate and presented the same dynamics of *Bcl2* family gene expression in testis and apoptosis of spermatogonia and spermatocytes [2].

In the last decade, chromium’s deleterious activity has been tried to be attenuated by introducing diverse natural antioxidants like curcumin [14], pycnogenol, a French maritime pine bark [15], garlic [16], *Spirulina platensis* [17], or extravirgin olive oil [18]. The incomplete information about the consequences of Cr(VI)’s oxidizing impact on suprarenal gland, and to identify if this impact can be altered by administering *A. melanocarpa L. aqueous extracts (as* phytotherapeutic mean to reduce/combat this), was the aim of this study*.*

## Materials and Methods

Approval for the experimental protocol (No. 60/2019) was obtained from the Ethical Committee of BUAVM Timișoara where the study was conducted.

### Animals

Thirty-six healthy male adult Wistar rats weighing 220–240 g were used. The animals were purchased from the University of Medicine and Pharmacy Timișoara, an authorized supplier. The rodents were kept for a 1-week acclimatization period in laboratory conditions at 22 ± 2 °C, temperature of 55 ± 10%, relative humidity, and respectively, 12 h light/dark cycle. The animals were fed with standard diet, Biovetimix code 140-501 (Biovet, Romania), and had free access to food and water. For housing, standard polycarbonate cages (l × w × h = 750 × 720 × 360 mm) were used.

### Chromium

The most water-soluble form of chromium salt, the potassium dichromate (K_2_Cr_2_O_7_) (*Sigma*, Germany), was used. Several studies, focused on chromium’s consequences on rat reproductive organs, evaluated some doses of Cr(VI) at 25 ppm, the dose confirmed by the International Agency for Research on Cancer as the lowest observed adverse effect level (LOAEL), 50 ppm (2 × LOAEL), and 75 ppm (3 × LOAEL) [19]. Based on obtained results, which highlighted that chromium that gives injuries is dose dependent, the 75 ppm Cr(VI) was chosen as the individual dose.

### Plant material and experimental design

*Aronia melanocarpa* berries were bought from a herbal store (DE-ÖKO-034-*Aronia Naturproduckte*, Germany) to obtain an extract. Berries were macerated in cold water at 0.25/10 water/volume proportion, then boiled at 90 °C for 10 min, and finally, the mixture was strained [20].

Rats were randomly divided into six groups of six animals, and group-specific treatments were applied as summarized in Table 1.

**Table 1:** Presentation of the studied groups

Group	Abreviation	Administration scheme	Exposure period
Control	C	Distilled water	3 months ad libitum
Chromium	Cr	Cr(VI)—75 ppm in distilled water	3 months ad libitum
Chromium + *Aronia*	CrA	Mixture of Cr(VI)—75 ppm in distilled water + *A. melanocarpa* 2.5% aqueous extract	3 months ad libitum
Chromium 2	Cr2	Cr(VI)—75 ppm in distilled water	3 months followed by 1 month with distilled water only ad libitum
Chromium + *Aronia* 2	CrA2	Cr(VI)—75 ppm in distilled water	3 months followed by 1-month with *A. melanocarpa* at 2.5% aqueous extract administration ad libitum
*Aronia*	Ar	*A. melanocarpa* aqueous extract	3 months ad libitum

After the exposure periods, rats were killed by anesthetic overdosing, using ketamine (Ketamine) (*CP Pharma*, Germany), and xylazine (Narcoxyl, *Intervet*, The Netherlands). The procedures through the study and euthanasia were conducted, following Directive 2010/63/EU, and the NRC Guidelines [21, 22].

### Histological examination

Adrenal glands were gathered and fixed for 18 h, in Bouin’s–Hollande solution. Then, samples were washed in distilled water and dehydrated by immersing in ethanol’s growing concentrations. Consequently, ethanol was replaced with xylene, and samples hardened in paraffin (*Merck*, Germany). Five-millimeter slices were sectioned, to a Cut-4062 microtome (Mainz, Germany), inserted on slides, and stained, using hematoxylin and eosin (H&E) and Mallory's trichrome techniques. Microscopy was made, to × 100, 200, and 400 magnification, to a CX41 microscope (*Olympus*, Germany), including digital camera and QuickPHOTO-Micro2.2. software (*Promicra*, Czech Republic), for the images’ interpretation.

### Real-time qRT-PCR

The total RNA was isolated and purified using 50 mg sample and the SV Total RNA Isolation System (Promega, USA). Purified RNA quality and quantity were determined with a NanoDrop 8000 spectrophotometer (Thermo Scientific, USA), and 2 μl total RNA samples were used for the cDNA synthesis, utilizing a cDNA First Synthesis Kit (Thermo Scientific, USA). As a template, in the qPCR experiments, 150 ng of cDNA was checked. For gene expression, the MX 3000P real-time PCR system (Agilent Technologies, USA) and GoTaq® qPCR Master Mix Kit (Promega, USA) were utilized. The primer sequences implied:

- Glyceraldehyde 3-phosphate dehydrogenase (GAPDH) reference gene (sense/antisense): ATGGAGAAGGCTGGGGCTCACCT/AGCCCTTCCACGATGCCAAAGTTGT;
- *Bax* target gene: CCAGGACGCATCCACCAAGAAGC/TGCCACACGGAAGAAGACCTCTCG;
- *Bcl2* target gene: GGATGACTTCTCTCGTCGCTACCGT/ATCCCTGAAGAGTTCCTCCACCAC.

Samples were run in triplicate, and the cycle threshold (Ct) values were normalized using the GAPDH gene expression C with the obtained results being interpreted by 2-ΔΔC (T) method [23].

### Statistical Analysis

All values were analyzed by one-way ANOVA, being expressed as mean ± standard error of means (SEM). The analysis was done using the Stata13 program (Stata Corp LP, USA). Statistical values considered are as follows: *0.01 ≤ *p* < 0.05, significant; **0.001 ≤ *p* < 0.01, highly significant; and ***p < 0.001, very high significant.

## Results

### Histological Assessment

**Fig. 1:**
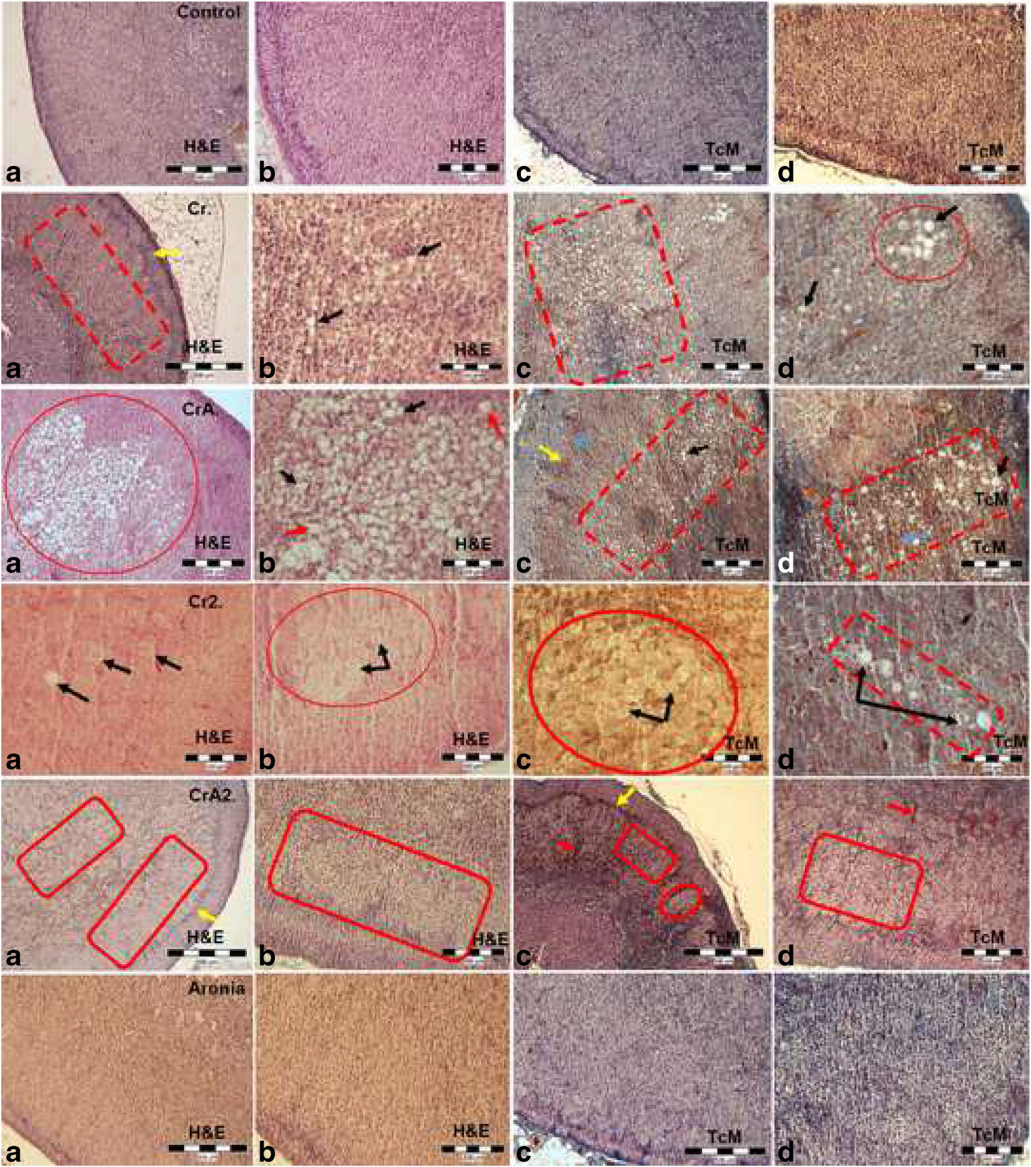
Adrenal citoarchitecture/studied groups

In *control* and *Aronia* groups, histological examination illustrated a normal structure of the adrenal gland. Structurally, it consists of two zones: an external cortex located under a thin capsule of the connective tissue and, an inner medulla, placed in the gland's core (Fig. 1 (1a–d)). The adrenal cortex, including various cells, is arranged in cords and split into three zones: *glomerulosa, fasciculata,* and *reticularis*. In rats, placed between *zona glomerulosa* and *fasciculata,* a fourth area, *zona intermediata,* containing small cells with no lipidic vesicles and considered as adrenal gland stem cells, was described [24].

In the *Cr group*, the presence of hypertrophied irregular-sized cells was revealed. Due to lipid droplet accumulation, some cells rebuilt their aspect, turning spherical, though greatest spongiocytes, although hypertrophied, preserved their polygonal shape (Fig. 1 (2b)). Diffuse hypertrophy was the dominant architectural change here (Fig. 1 (2a, c)), but focal hypertrophy was also identified (Fig. 1 (2d)). The hypertrophied spongiocytes displayed vacuolar cytoplasm occupied by lipidic droplets of different dimensions, explaining why it is so poorly stained, and cells’ faded look. Nuclei were prominent, spherical, and centrally grouped, but in the lipid-filled cells, the nuclei migrated eccentrically. Bi-nucleated spongiocytes were also seen.

In the *CrA* group, the cells from *zona glomerulosa* exhibited a polygonal shape but, more extended and larger, than spongiocytes of adjacent areas (Fig. 1 (3c)). The nuclei, delimited and spherical, kept them in central position, and the cytoplasm was abundant, basophilic, with small vacuoles; capillaries separating the cell cords were also noticed (Fig. 1 (3d)). Cells’ focal (Fig. 1 (3a, b)), and/or diffuse (Fig. 1 (3c)) hypertrophy, in *zona fasciculata,* was perceived. Here, the spongiocytes were hypertrophied and the cytoplasm is filled with numerous vacuoles, whose volume and fullness determine the cells’ shape from polygonal to spherical. The cells filled with small vacuoles, or with a single large one, were recorded, giving the cytoplasm’s tone from slight acidophilic to pale. The nuclei were delimited, expanded, and spherical shaped, though, in the hypertrophied cells, these are shifted. In focal hypertrophy, lipidic degeneration was observed, as well as necrosis and dilatation of the fenestrated capillaries.

In *zona reticularis,* most of the cells presented normal appearance and a stained cytoplasm in contrast with the spongiocytes from *zona fasciculata,* which indicate the cytoplasmic vacuoles’ absence.

In *the zona glomerulosa of the Cr2 group,* hypertrophied polygonal cells, larger than those from the *control* and *Aronia* groups, were recognized. Distended capillaries separated the cellular cords and cytoplasm of hypertrophied cells was abundant and clear, including numerous vacuoles. The nuclei were spherical, well highlighted, and centrally positioned.

The hypertrophied cells of the *zona fasciculata* were, both, dispersed (Fig. 1 (4d)), or, grouped in nests (Fig. 1 (4b, c)). The dispersed cells were of different spherical sizes, with abounding cytoplasm, appearance due to accumulated lipid droplets, the vacuoles being, both, small and numerous, or large and rare. Well-defined, spherical-shaped, and centrally arranged nuclei were observed. Grouped hypertrophied cells, and bi-nucleated spongiocytes, were also noted (Fig. 1 (4a)). In the *zona reticularis*, the majority of cells had a normal appearance with a stained cytoplasm, in contrast with spongiocytes of *zona fasciculata*.

In *the CrA2 group*, diffuse cytoplasmic vacuolization affected the adrenal cortex, and mostly, the spongiocytes (Fig. 1 (5a, d)). The vacuoles in the cytoplasm were small, if they were many, or large, if they were a few. Cells were differentiated, polygonal, or spherical and separated by the widened capillaries (Fig. 1 (5c, d)). Capillaries’ ectasia, although present in the adrenal cortical parenchyma of the *zona fasciculata, was observed.* The cytoplasm abounding in vacuoles was poorly stained. Nuclei were large, spherical, and centrally positioned.

Cells from the *zona reticularis* presented a normal appearance and just limited hypertrophied cells (Fig. 1 (5a, c)); the capillaries’ ectasia was also recorded.

In all groups, the *zona intermediate* was well represented, including a large cell population (Fig. 1 (2a, 3c, 4c, 5a, 5c)).

### Molecular assessment

The *Bax* proapoptotic gene expression levels revealed a significant increase (*p* < 0.001) in all groups compared with the *control,* being perceived as important variations within groups. The most prominent gene expression was reported in *the Cr* group, exposed for 3 months to CrVI. In the *Cr2* group, notable reduction of *Bax* gene expression (*p* < 0.001) was reported compared with the *Cr* group.

Administration of *A. melanocarpa*, in *the CrA2* group, decreased the *Bax* gene expression, compared with the *Cr group.* In the case of joint administration of CrVI and *Aronia* (*CrA*), a significant reduction of *Bax* gene expression (*p* < 0.001), compared with *the Cr group, was ascertained.* The variation within the *CrA* and *control, or Aronia* groups, although with little value, it was present, and statistically significant (*p* < 0.001), validating the CrVI’s exerted cellular stress. A significant increase of *the Bcl2* gene expression (*p* < 0.001) was observed in the experimental groups, compared with the *control*. The *Bcl2* expression level had a similar pattern to *the Bax* gene, consequently trying to compensate its overexpression.

**Fig. 2:**
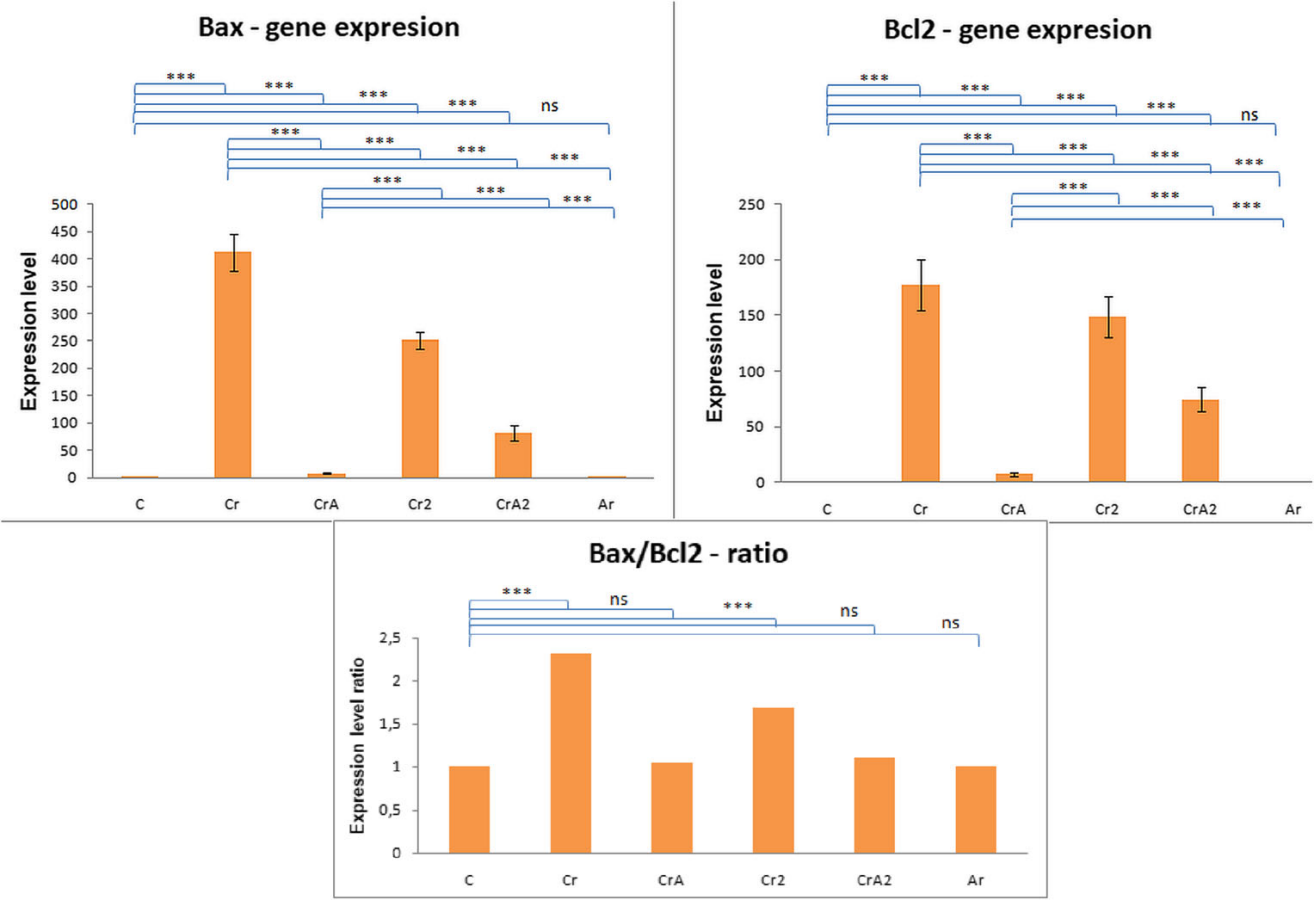
Expression of proapoptotic and antiapoptotic genes and them signification in adrenal gland: **a** *Bax* gene expression (***p < 0.001); **b** *Bcl2* gene expression (***p < 0.001); **c** *Bax/Bcl2* ratio

The *Bax/Bcl2* ratio (Fig. 2) indicates the organism’s capability to adapt to chromium’s adverse action, or even to overcome it, also revealing the *Aronia’s* effectiveness.

In the *Cr* group, the *Bax/Bcl2* ratio is in favor of the *Bax* gene (*Cr Bax/Bcl2-2.32)*, emphasizing that the cells are subjected to continuous oxidative stress, of high intensity, for a long time, so that the adaptive mechanisms of the body can be overcome, all of which can lead to the triggering of the apoptotic process. In this *Cr2* experimental group, the *Bax/Bcl2* ratio in favor of the *Bax* gene (*Bax/Bcl2-1.68)* suggests that the cells were subjected to high-intensity oxidative stress, but due to the post-toxic recovery period, the adaptation mechanisms partially compensate for the toxic actions.

In the *CrA* and *CrA2* groups, the *Bax/Bcl2* ratio, around value 1 (*CrA Bax/Bcl2-1.04; CrA2 Bax/Bcl2-1*), is close to that of the control group, suggesting that the active ingredients present in *Aronia*, which were administered either concomitantly with or after potassium dichromate, are capable of blocking the apoptotic cascade. This fact is due to the active compounds of aqueous extract co-administered with potassium dichromate, capable of reducing induced oxidative stress. In the case of the *CrA2* group, the beneficial effect of the aqueous extract of *Aronia*, which was administered for 1 month but only after the treatment with potassium dichromate was stopped, is cumulated with the recovery period, in which the toxicity was absent.

## Discussion

Apoptosis is a “programmed cell death” providing, along with the cell proliferation, architectural and functional homeostasis. The connection between apoptosis and cells’ proliferation ensures the organs’ modification and the continuance of each cell type, by excluding the abnormal ones. Apoptosis is described as the whole-cell deterioration, accompanied by its fragmentation in apoptotic bodies, which swiftly, are excreted from the body. The intracellular content is separated by these apoptotic bodies so that the inflammatory response does not occur. Due to this fact, and the speed of this process, apoptosis detection is a challenging process [25].

The proteins from *the Bcl2* family, especially the *Bax/Bcl2* ratio, regulate the execution phase of apoptosis controlling the mitochondria. The excess of *Bcl2* (antiapoptotic), assures a cell’s survival, either by direct blocking of *Bax* protein action or by preventing the caspases activation (the main enzymes involved in apoptosis), influencing the mitochondrial membrane permeability [2, 3]. Excess of *Bax* (proapoptotic) leads to the *Bcl2* inactivation and changes in mitochondrial transmembrane potential, through the formation of transmembrane complexes. As a result, exchanges between the mitochondrial compartment and cytoplasm will escalate, leading to the release of cytochrome *c* proteins, SMAC/DIABLO, which will generate caspases activation and triggers the apoptotic cascade [11].

Studies have shown that once inside the cell, Cr(VI) is reduced to Cr(III), a process during which the intermediate forms; Cr(V) and Cr(IV), and also various ROS are produced. Their direct binding to cellular constituents may explain the cytotoxicity of chromium, manifested by interruption of the cell cycle, neoplastic transformation, or apoptosis induction [26]. Exposure to CrVI can lead to DNA replication and transcription dysfunction, dysregulation of DNA repair mechanisms, microsatellite instability, inflammatory responses, and impairment of genes responsible for maintaining the cell survival/death balance [27]. Thus, hexavalent chromium can produce chromosomal aberrations, changes between sister chromatids, DNA strand breaks, base oxidation, the formation of Cr-DNA, DNA-Cr-DNA, or protein-Cr-DNA compounds, either directly, or indirectly, through ROS production [28].

The adrenal gland is one of the most susceptive endocrine organs to the toxicants [29], but no extensive knowledge yet, about the intimate effects of Cr(VI), on the adrenal structure and function. Adrenal vulnerability to the environmental pollutants is due to its properties, like the high content of unsaturated fatty acids in the cell membrane. These are highly susceptible to the lipidic peroxidation, directly, by the activity of compounds or metabolites, or indirectly, by the free radicals’ presence. The P450 enzymes, on the other hand, are accountable for the production of the reactive metabolites, and of mediating toxicity; also, hydroxylation can produce dangerous free radicals for the cell membranes. High vascularity and lipophilicity (the high cholesterol and steroid content) also contributes to the adrenal susceptibility to toxicants [30]. This can explain one outcome of our investigation, that the adrenal glands’ cytoarchitecture was altered after the administration of Cr(VI).

Results for the *Cr* and *Cr2* groups revealed a high level of the *Bax* gene. In a compensatory way, we also observed a significant increase in the *Bcl2* gene expression, *the Bax/Bcl2* ratio, being in the *Bax* gene favor in these groups, and indicating that the adrenal cells were subjected, for a long time, to high-intensity oxidative stress. This affirmation is validated by the observed structural alterations, in all areas of the adrenal cortex like diffuse hypertrophy, cytoplasm vacuolization, presence of different size lipidic droplets, describing degeneration, and the presence of the binucleated cells. In the *Cr2 group*, although these genes were well displayed, compared with *the Cr group,* their level was lower, because the individuals from this *group* availed from 1-month recovery, after the interruption of the CrVI administration. Low values were registered, for groups, *CrA2, control, and Aronia, with the balance in favor of the Bcl2* gene, confirming the *Aronia’s* efficiency and reflected by the observed adrenal cytoarchitecture.

Although the effect of chromium on the adrenal gland was not excessively studied, some studies presented the influence of diverse toxicants on this tissue. Latendresse et al. reported comparable vacuolar degeneration in rat’s adrenal gland, following the administration of organophosphates [31].

Toxicants that can induce severe adrenal vacuolar degeneration, often lead to necrosis, cell lysis, or apoptosis of adrenocortical cells. Nishimura et al. reported histological changes of *zona fasciculata*: vacuolation, hypertrophy, and degeneration of the cells after acetyl-coenzyme A:cholesterol *O*-acyltransferase inhibitor administration [32].

Pereira et al. observed that polychlorinated biphenyls and diethyl phthalate produce intracellular vacuolations and degeneration in *zona fasciculata.* They assumed that this fact could bring impairment in the synthesis and secretion of corticosterone, and thus, loss of steroidogenesis in the adrenal gland [33].

Therefore, steroids are involved in diverse processes, and consequently, a balance between steroid production in the gonads, and the endocrine glands, have to be maintained to prevent critical physiological outcomes. Therefore, the presented architectural changes of the adrenal cortex could lead to an imbalance in the levels of the steroids, which can be a cause of reproductive capacity impairment, previously described in studies about the consequences of hexavalent chromium on male’s reproductive tract [34, 35].

The results confirm that, with the suspension of chromium administration, the *Bax* and *Bcl2* genes expression levels declined, confirming that the adaptation mechanisms started to recover, by compensating (just partially), the toxicant’s action. Though, the reported lesions of the adrenal cortex have not disappeared entirely, prompting us to think that the interval needed for the cellular structure’s recovery was longer.

The active compounds from berries of *A. melanocarpa,* responsible for therapeutic effects, are represented by its phenolic nature compounds. Among all other blackberries, *A. melanocarpa* holds the highest quantity of phenolic compounds, certainly, correlated with its high antioxidant activity [7]. These compounds act as main hydrogen donors, oxygen quenchers, and reducing agents due to the redox properties [36].

Mężyńska et al. demonstrated the antioxidant properties of *A. melanocarpa L. extract against cadmium-induced oxidative stress and structural changes in liver, suggesting that, due to the special composition, high amount of polyphenols, the Aronia is able to protect organs and tissues against oxidative stress produced by xenobiotic substances with pro-oxidant properties* *[*37*].*

Whereas anthocyanins possess the ability to protect the cells against apoptosis [38], Meng et al., by using anthocyanins from *A. melanocarpa L. showed* that apoptosis can be attenuated by inhibition of mitochondrial dysfunction via increasing *Bcl2* and decreasing *Bax* protein expression [8].

Accordingly, *A. melanocarpa* was used to overcome the Cr(VI) oxidative consequences. For instance, in the *CrA group,* the *Bax* and *Bcl2* gene expression remained feebler, related to experimental groups, being closer to *the control group.* In *the CrA2 group*, we learned the two genes’ overexpression, however, is feebler than for *the Cr group*. In both cases, the *Bax/Bcl2* ratio (around = 1) was closer to the control. This confirms that the *Aronia’s* active constituents, given concomitantly, or following Cr(VI), overcame, in a considerable measure, the oxidative stress, controlling the apoptosis process regulated by *Bax/Bcl*. The deleterious impact of chromium was visible, altering the adrenal cytoarchitecture, especially in the groups exposed to Cr(VI), given an extended time. Alternatively, in the *zona reticularis*, cells with healthy characteristics were preponderant. Adrenal cells are incorporating lipids and cholesterol in their cytoplasm. Regularly, the healthy cells hold these lipids, in low quantities in their cytoplasm, but excessive accumulation may alter the cell’s structure and functionality [39].

Cortical hypertrophy, of the *zona fasciculata*, appears to be a consequence of the increased level of adrenocorticotropic hormone (ACTH), increase that can be produced by different causes: disorders of the hypothalamus or the pituitary gland, defective regulation of glucocorticoid secretion, and toxic lesions at adrenocortical level [30], as those described in our study. According to the studies, the vacuolization of cells from the adrenal cortex can be the biological response of the gland to stress and the continuous endogenous stimulation of ACTH. Another cause may be the administration of drugs and toxins, those that interfere with the hypothalamic-pituitary-adrenal axis [29, 39], moreover, a demonstrated action of chromium [40].

Impaired secretion of pituitary hormones may increase the vulnerability of the endocrine glands to chromium. As studies have shown, the pituitary gland and hypothalamus are also affected by hexavalent chromium which accumulates at this level and induces oxidative stress and apoptosis. Therefore chromium, and especially Cr(VI), has negative impacts in the endocrine function [40, 41].

Also, exposure to Cr(VI) could increase the adrenal’s activity. Activation of the hypothalamic-pituitary-adrenal axis is followed by excessive synthesis and release of glucocorticoids, to reduce the stress influence [1]. All this originates from the hypothalamus, which under the stress effect, secretes massive amounts of corticotropin-releasing factor (CRH), which will stimulate the secretion and release of ACTH by the pituitary gland. This hormone works on the adrenal gland, stimulating the glucocorticoid hormone secretion, which may explain, the presence of a large quantity of lipid-filled cytoplasmic vacuoles, also observed by us, required for the synthesis of the hormones. Although the endocrine cells from the *zona reticularis* are producing androgen hormones in rats, they are also responsible for the glucocoticoid hormones’ synthesis in small amounts [24]. Considering this, we can assume that the structural changes represented by the lipidic accumulation in the cytoplasm, appear due to the stimulation of the endocrine cells from zona reticularis caused by Cr(VI) in hypothalamic-pituitary-adrenal axis.

According to the literature, the toxic effect of hexavalent chromium is exhibited by the production of free radicals, thus increasing cellular oxidative stress, which can induce the apoptotic process [42]. Taking into account the installation of the apoptotic process, we can evaluate the state of this process thereof by monitoring the *Bax/Bcl2* ratio [43].

According to the literature consulted, oxidative stress is a key player in Cr(VI)-induced toxicity, and even though the expression profiles of the caspases related to the apoptosis could have provided a broader view of the study, the aim in this stage of research was only the evaluation of Bax/Bcl2 ratio, which it is known, that indirectly mirrors the oxidative stress rate, and the induction of the apoptosis phenomenon.

The oxidative stress is a key player in the Cr(VI)-induced toxicity. Therefore, it is possible to monitor, indirectly, the increase of oxidative stress, due to the toxic action of hexavalent chromium at the cellular level, by evaluating the *Bax/Bcl2* ratio, the ratio resulting from the direct actions of ROS produced by chromium.

## Conclusion

One important finding of this research is that Cr(VI) administration in rats induces morphological changes of the adrenal gland, most probably because of ROS production following oxidative stress induction. Treatments with *A. melanocarpa L.* aqueous extracts provide important protection of the adrenal glands during architectural alterations, and consequently, these extracts may be used as a hexavalent chromium-adrenal toxicity suppressant, as the *Bax/Bcl*2 ratio revealed.
